# Acute inflammation upregulates FAHFAs in adipose tissue and in differentiated adipocytes

**DOI:** 10.1016/j.jbc.2024.107972

**Published:** 2024-11-05

**Authors:** Meric Erikci Ertunc, Srihari Konduri, Zhichen Ma, Antonio F.M. Pinto, Cynthia J. Donaldson, Jeremiah Momper, Dionicio Siegel, Alan Saghatelian

**Affiliations:** 1 Clayton Foundation Laboratories for Peptide Biology, The Salk Institute for Biological Studies, La Jolla, California, USA; 2 The Skaggs School of Pharmacy and Pharmaceutical Sciences, University of California San Diego, La Jolla, California, USA

**Keywords:** inflammation, lipid, LPS, adipose tissue, ATGL, FAHFA, HODE, HETE, LTB4

## Abstract

Since the discovery of fatty acid hydroxy fatty acids (FAHFAs), significant progress has been made in understanding their regulation, biochemistry, and physiological activities. Here, we contribute to this understanding by revealing that inflammation induces the production of fatty acid hydroxy stearic acids and fatty acid hydroxyoctadecadienoic acids in white adipose tissue depots and in adipocytes cocultured with macrophages. In lipopolysaccharide (LPS)-induced coculture systems, we confirm that adipose triglyceride lipase is required for inflammation-induced FAHFA generation and demonstrate that inflammation is necessary for producing hydroxy fatty acids. Chemically synthesized fatty acid hydroxyoctadecadienoic acids show anti-inflammatory activities *in vivo*, but only at supraphysiological concentrations. While endogenous FAHFAs are unlikely to be anti-inflammatory due to their low concentrations, conversion of proinflammatory hydroxy fatty acids into FAHFAs may dampen inflammation. Indeed, we demonstrate that proinflammatory lipids, such as hydroxyeicosatetraenoic acids (HETEs) and leukotriene B4 (LTB4), can be converted by cells in culture to weakly anti-inflammatory FAHFAs.

Fatty acid esters of hydroxy fatty acids (FAHFAs) are a class of lipids discovered as being highly upregulated in the adipose tissue from adipose-specific glut 4 overexpressing mice (1). They are classified into families based on their acyl chain composition, and each family has multiple isomers that are denoted by the branch point of the ester (Fig. 1, and Table S1). Since their discovery, hundreds of FAHFA families and their corresponding isomers have been identified in animals, plants, and yeast (1, 2, 3, 4, 5, 6, 7). Pharmacological studies with FAHFAs demonstrated anti-inflammatory and immunomodulatory activity in animal models (1, 8, 9, 10, 11) and in cell culture (adipocytes and immune cells) (1, 4, 8, 10).

**Figure 1:**
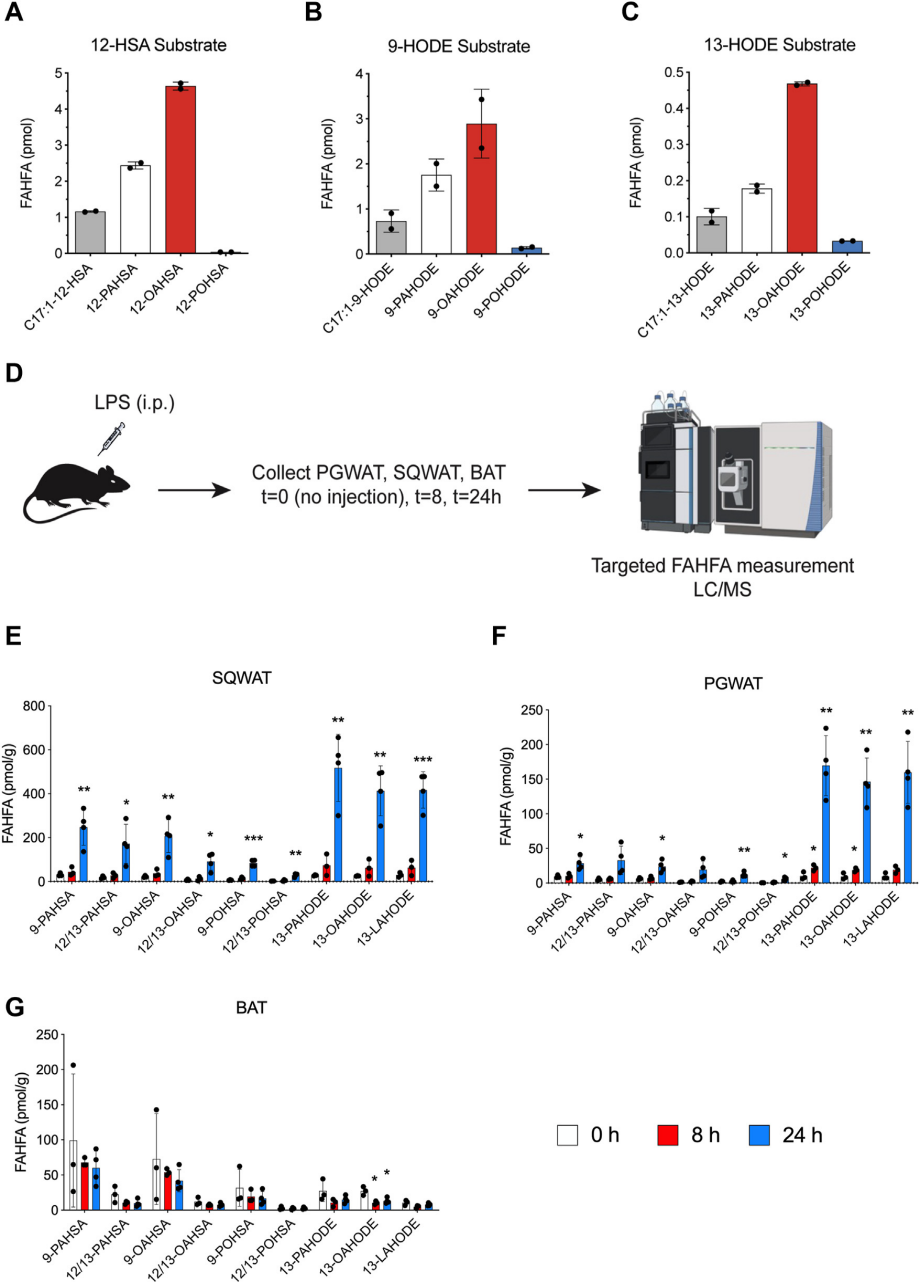
**HSA and HODE derived FAHFAs are upregulated in adipose tissue upon LPS treatment *in vivo*.** RAW cells were treated with 100 μM (*A*) 12-HSA, (*B*) 9-HODE, or (*C*) 13-HODE along with 20 mM C17:1 for 2 h. HSA and HODE-derived FAHFAs were measured *via* targeted LC-MS (n = 2 per group). *D*, experimental scheme for (*E*–*G*). Mice were injected with 3 mg/kg LPS i.p. Tissues were collected at t = 0 h, 8 h, and 24 h. FAHFAs in (*E*) PGWAT, (*F*) SQWAT, and (*G*) BAT were measured *via* targeted LC-MS (n = 3–4 per group. ∗, *p* < 0.05; ∗∗, *p* < 0.01; and ∗∗∗, *p* < 0.001, compared with t = 0. *t* test). Error bars represent SD. FAHFA, fatty acid hydroxy fatty acid; HETE, hydroxyeicosatetraenoic acid; HODE, hydroxyoctadecadienoic; i.p., intraperitoneally; LPS, lipopolysaccharide.

In search of enzymes that regulate FAHFAs, few FAHFA hydrolases were discovered *in vitro* such as androgen-induced gene 1, androgen-dependent tissue factor pathway inhibitor-regulating protein, and carboxyl ester lipase (12, 13) as well as *in vivo* such as androgen-induced gene 1 and androgen-dependent tissue factor pathway inhibitor-regulating protein (14). Peroxiredoxin-6 and glutathione peroxidase 4 were implicated in FAHFA biosynthesis (15). Adipose triglyceride lipase (ATGL) has two roles in regulating FAHFA levels including as the primary FAHFA biosynthetic enzyme in adipose. ATGL was first noted to hydrolyze triglyceride-containing FAHFAs (16). More recently, ATGL was discovered to have a transacylase activity that transfers an acyl chain from a triglyceride to a hydroxy fatty acid to produce the ester bond found in all FAHFAs (17).

As mentioned, FAHFAs were discovered because of their marked upregulation in adipose-specific glut 4 overexpressing mice (>10-fold). Additional measurements showed modest regulation during feeding-fasting cycles and high-fat diet (1), and separate study looking at cold exposure after 7 days at 6 °C found increased levels in palmitic acid ester of 9-hydroxystearic acid (9-PAHSA) when compared to thermoneutral mice in white adipose tissue (18). While FAHFAs have anti-inflammatory activity *in vitro* and *in vivo*, to our knowledge their regulation following an acute inflammatory insult has not been studied directly.

Here, we utilized cellular assays and mouse models to discovery that FAHFAs are upregulated *in vivo* and *in vitro* upon LPS induction of inflammation, and that this upregulation includes FAHFAs that contain a 13-hydroxyoctadecadienoic acid (13-HODE), a known inflammatory marker, as the hydroxy fatty acid. Cellular assays demonstrated that inflammation leads increased hydroxy fatty acid levels, which is unsurprising, and these hydroxy fatty acids are then converted into FAHFAs through the transacylation activity of the known biosynthetic enzyme ATGL.

As with other FAHFAs, we synthesized and tested fatty acid esters of hydroxyoctadecadienoic acids (FA-HODEs) and found that they had modest anti-inflammatory *in vivo* and *in vitro*, but only at supraphysiological concentrations. We reasoned that while these lipids might not have anti-inflammatory activity at physiological concentrations, the conversion of proinflammatory hydroxy fatty acids, such as HODEs, into FAHFAs might indicate an unappreciated biochemical pathway to modulate inflammation. We tested this possibility *in vitro* and observed that proinflammatory hydroxy fatty acids, including the leukotriene B4 (LTB4), are readily converted into FAHFAs. In total, this work reveals that FAHFAs, especially fatty acid HODEs, are regulated in adipose tissue after LPS treatment, and suggest that this pathway may regulate inflammation *via* the acylation of proinflammatory hydroxy fatty acids, with LTB4 being an exemplar.

## Results

### LPS treatment of mice upregulates FAHFAs over 24 h

FAHFAs are be synthesized *via* esterification of fatty acids and hydroxylated fatty acids mediated by ATGL (17). Inflammation is known to promote lipid oxidation to generate a number of hydroxy fatty acids, including HODEs from the oxidation of linoleic acids, and hydroxyeicosatetraenoic acids (HETEs) and leukotrienes from the oxidation of arachidonic acid. We focused on HODEs to test the idea that inflammatory conditions that oxidize fatty acids will also lead to an increase in FAHFA levels, because HODEs are the most abundant of the aforementioned lipid classes and therefore the easiest to detect (19).

We began by testing whether the addition of HODEs to cells resulted in FAHFA production. Treatment of RAW 264.7 cells were treated with heptadecenoic acid (C17:1), along with a HODE or a hydroxy stearic acid resulted in the production of heptadecenoic FAHFAs containing HODEs and hydroxystearic acids (Fig. 1*A*). These experiments confirmed that mammalian cells can use a variety of different HFAs to produce FAHFAs and provide the rationale for testing to see whether proinflammatory conditions that generate oxidized lipids (*i*.*e*., HODEs (20, 21)) might also produce FAHFAs *in vivo* (Fig. 1, *A–C*).

**Figure 2:**
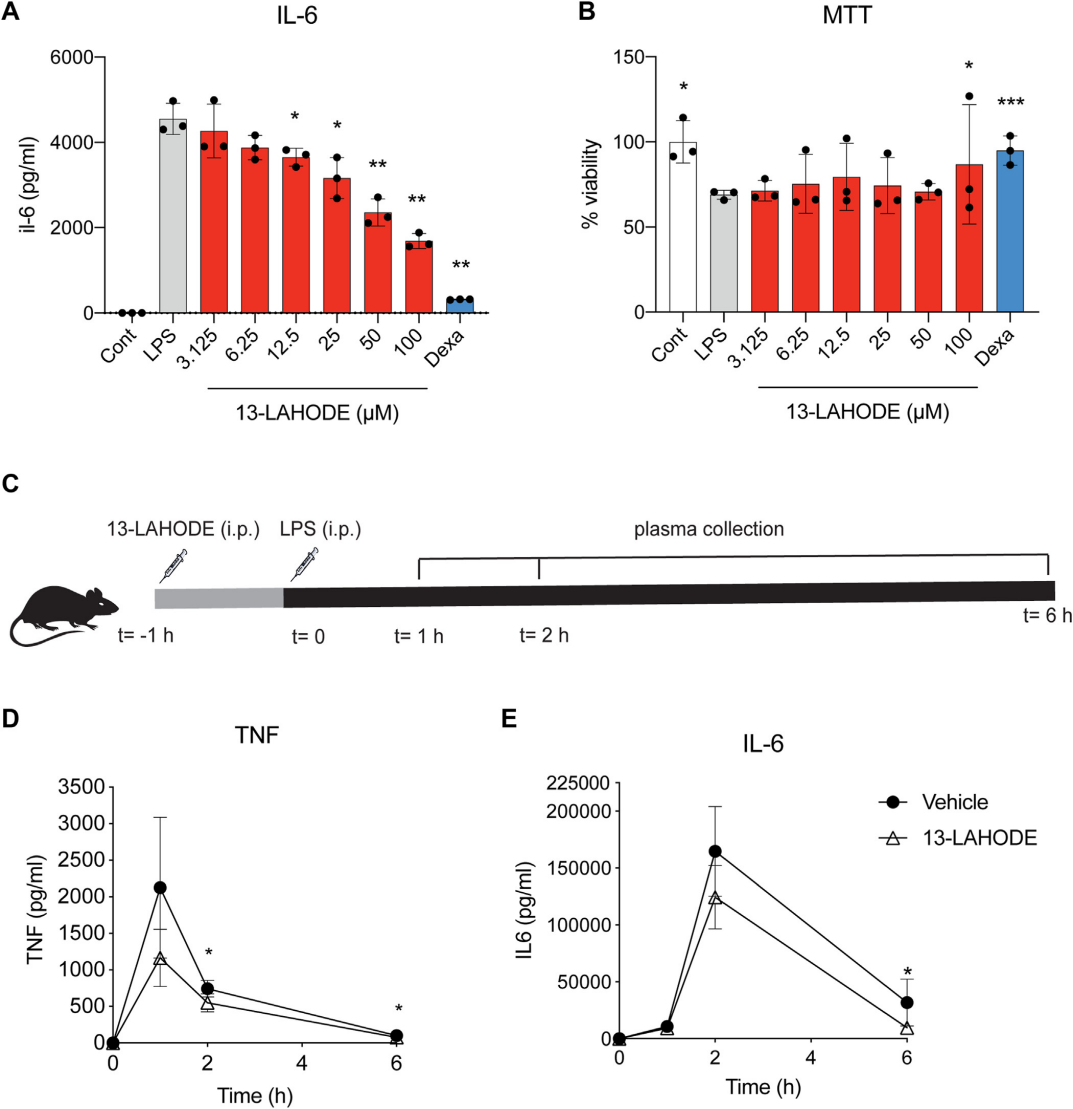
**13-LAHODE has anti-inflammatory activity *in vivo*.** RAW cells were treated with vehicle (control), or 100 ng/ml LPS with or without 5 μM dexamethasone or indicated concentrations of 13-LAHODE for 24 h. *A*, IL-6 levels were measured *via* ELISA. *B*, cell viability was inferred using MTT assay (n = 3 per group. ∗, *p* < 0.05; ∗∗, *p* < 0.01; and ∗∗∗, *p* < 0.001, compared with LPS. *t* test). *C*, experimental scheme for *in vivo* anti-inflammatory assay. Mice were injected with 10 mg/kg 13-LAHODE or vehicle at t = −1 h. Then, injected with 0.15 mg/kg LPS. Plasma samples were collected at t = 0, 1 h, 2 h, and 6 h. Plasma (*D*) TNF and (*E*) IL-6 levels were measured by ELISA (n = 5 per group. ∗, *p* < 0.05, compared with vehicle. *t* test). Error bars represent SD. 13-LAHODE, linoleic acid ester of 13-HODE.

Mice fed a chow diet were treated with LPS intraperitoneally (i.p.) and FAHFAs containing fatty acid hydroxy stearic acids and FAHODEs from perigonadal white adipose tissue (PGWAT), subcutaneous white adipose tissue (SQWAT), and brown adipose tissue (BAT) by LC-MS at 0, 8, and 24 h post-LPS administration. The data demonstrated a continued increase in fatty acid hydroxy stearic acid and FAHODE levels over 24 h, with increases of ∼6- to 18-fold in SQWAT, and ∼3- to 15-fold in PGWAT (Figs. 1, *E* and *F*, and 2, *e*. *g*., LC-MS chromatograms). Unlike *in vitro* experiments where we supplemented HODEs, the dramatic increase in FAHODE levels *in vivo* is solely the result of LPS administration and reflects endogenous biochemistry. We did not observe similar increases in BAT (Fig.1*G*).

### 13-LAHODE has anti-inflammatory activity *in vivo*

The linoleic acid ester of 13-HODE (13-LAHODE) was selected for further experimentation since it was one of the most abundantly increased FAHFAs after LPS treatment and we had previously synthesized this lipid, which we detected in plant oils (22). We confirmed the anti-inflammatory of this batch of 13-LAHODE using an *in vitro* assay with LPS-treated RAW 264.7 mouse macrophage cells (Fig. 2, *A* and *B*). The dosing was inferred based on previously published acute and chronic FAHFA treatments in mice (9, 11). Mice were treated with 13-LAHODE 1 h prior to the administration of LPS, and plasma IL-6 and TNFα levels were measured at different time points using ELISA (Fig. 2*C*). 13-LAHODE–treated mice had a small decrease in circulating tumor necrosis factor alpha (TNFα) levels at 2- and 6-h post LPS injection (Fig. 2*D*) and interleukin-6 (IL-6) levels at 6 h post LPS injection (Fig. 2*E*). Together the data suggests that 13-LAHODE has mild anti-inflammatory activity *in vivo*. However, given the IC50 activity of this lipid *in vitro* is ∼12.5 to 25 μM and the endogenous concentrations of all the FAHFAs in adipose tissue might add up to 1 μM, the physiological relevance of this activity is doubtful.

### Crosstalk between immune cells and adipocytes is required for FAHFA production

**Figure 3:**
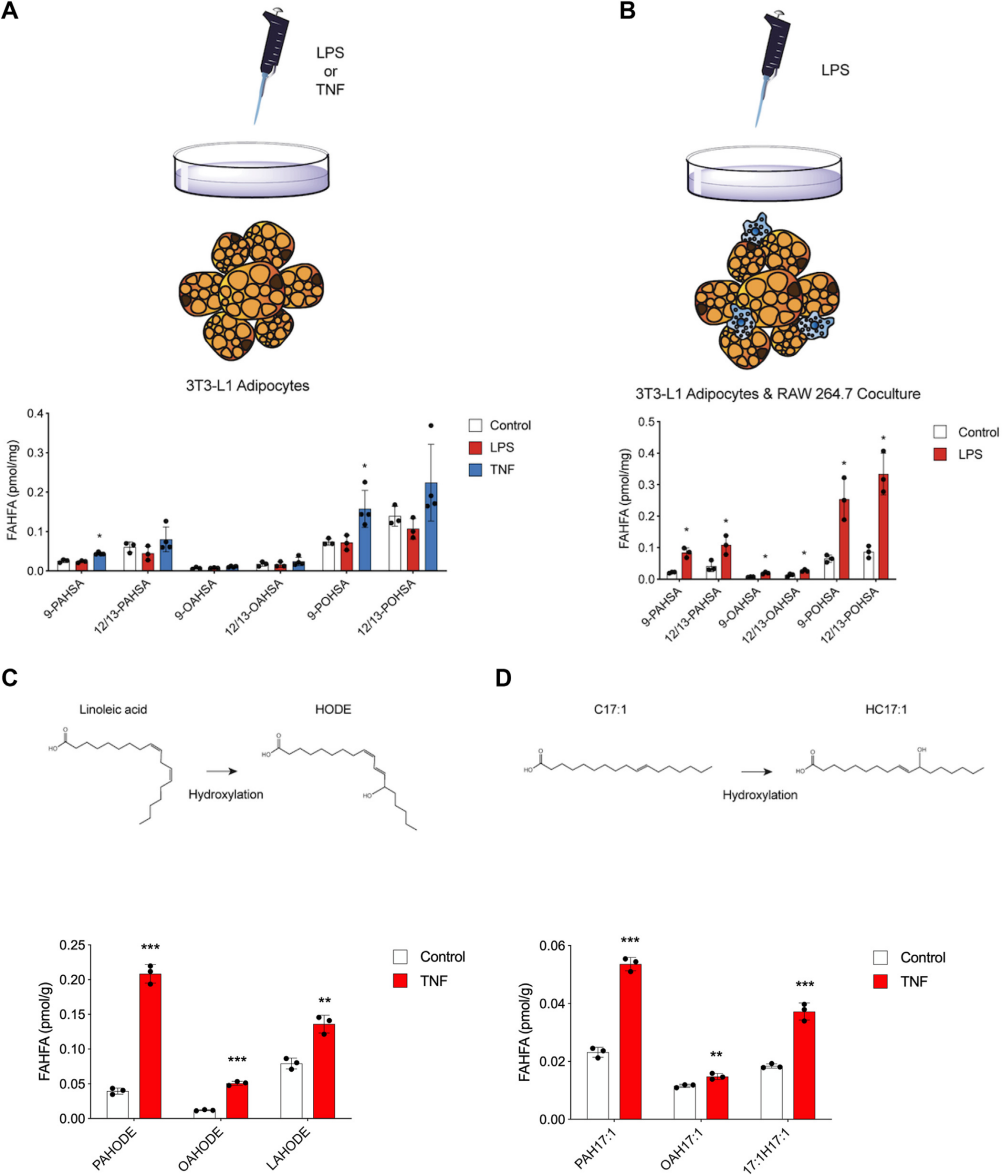
**FAHSAs and FAHODEs are upregulated upon inflammatory stimuli *in vitro*.***A*, 3T3-L1 adipocytes were treated with control media, 20 ng/ml TNF or 1 μg/ml LPS for 24 h. *B*, 3T3-L1 adipocytes cocultured with RAW cells were treated with control media or 100 ng/ml LPS for 24 h. FAHFA levels were measured using targeted LC-MS. *C*, *top panel*: conversion of linoleic acid to HODE *via* hydroxylation. *Bottom panel*: 3T3-L1 adipocytes were treated with control media or 20 ng/ml TNF for 22 h. One hundred micromolars linoleic acid was added and incubated for another 2 h. *D*, *top panel*: conversion of C17:1 (HA) to HC17:1 *via* hydroxylation. *Bottom panel*: 3T3-L1 adipocytes were treated with control media or 20 ng/ml TNF for 22 h. One hundred micromolars C17:1 was added and incubated for another 2 h. FAHFA levels were measured using targeted LC-MS. (n = 3–4 per group. ∗, *p* < 0.05; ∗∗, *p* < 0.01; and ∗∗∗, *p* < 0.001, compared with control. *t* test). Error bars represent SD. FAHFA, fatty acid hydroxy fatty acid; FAHODE, fatty acid hydroxyoctadecadienoic acid; FAHSA, fatty acid hydroxy stearic acid; HODE, hydroxyoctadecadienoic; HSAs, hydroxystearic acids.

To test if inflammation dependent upregulation of FAHFAs in adipose tissue can be recapitulated *in vitro*, we tested FAHFA production cell culture experiments (Fig. 3, *A* and *B*). Treatment of mouse 3T3-L1 differentiated adipocytes were treated with LPS for 24 h did not result in an increase in FAHFA levels, but the addition of TNF resulted in a small but significant increase in 9-PAHSA and palmitoleic acid ester of 9-hydroxystearic acid (9-POHSA) (Fig. 3*A*). This modest change suggested that we were missing a critical component of FAHFA production, and we looked to test FAHFA production in a coculture system where we would 3T3-L1 adipocytes were cocultured a mouse macrophage cell line (RAW276.4) and then treated with LPS for 24 h. Under the coculture conditions we find a robust increase in FAHFA levels (Fig. 3*B*), indicating that FAHFA regulation is due to crosstalk between the macrophages and adipocytes. We do not observe increases in FAHFAs at earlier time points or when macrophages were treated with LPS (Fig. S3), indicating the adipocyte is the primary FAHFA-producing cell. This is a highly interesting result and we tried to determine if this was due to secreted factor. However, treatment of 3T3L1 adipocytes with conditioned media (CM) from LPS induced macrophages had no impact on FAHFA levels (Fig. S4). This negative result suggests that FAHFA production requires contact between adipocytes and macrophages or that the secreted factor is unstable and does not collect in CM. The identification of this factor(s) would be of interest, but because of the open ended nature of these types of endeavors we focused on other questions.

### Inflammation-induced FAHODE production requires linoleic acid in cell culture

In the aforementioned experiments, we did not observe the upregulation of any FAHODEs. We reasoned that serum provides lipids to cells in culture but linoleic acid, the precursor to HODEs might be too low in concentration when using 10% serum-containing media. To test this and determine if cultured adipocytes can produce FAHODEs from linoleic acid, we repeated these experiments with linoleic acid (HODE precursor) after addition of TNF. The addition of linoleic acid led to the production of FAHODEs that are increased in the presence of TNF, which indicates that cultured adipocytes are able to produce and regulate FAHODE levels during inflammation (Fig. 3*C*). The data support a model where the addition of TNF leads to linoleic acid oxidation, followed by conversion to an FAHODE. To test whether any fatty acid could be used in this pathway, we repeated this experiment with the heptadecenoic acid, a minor component of fat, and observed the successful incorporation of this lipid into FAHFAs (Fig. 3*D*), which is supportive of general lipid oxidation that is associated with inflammation.

### ATGL mediates inflammation-induced FAHFA biosynthesis

**Figure 4:**
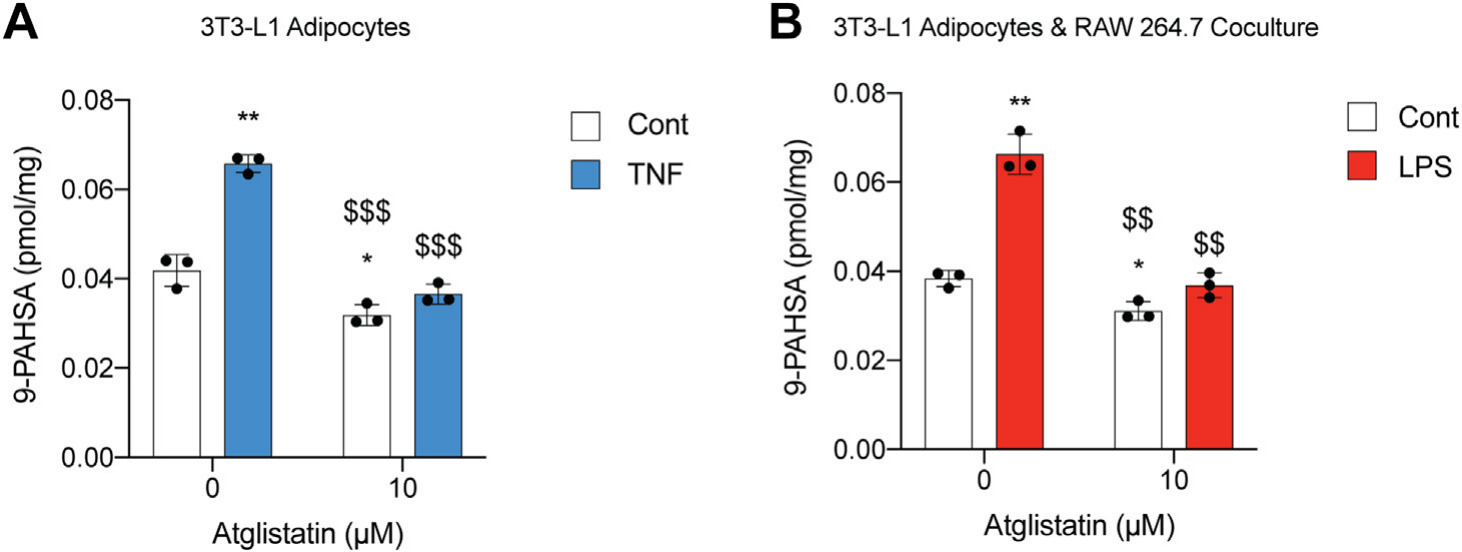
**Inflammation induced upregulation of FAHFAs is inhibited by ATGL inhibitor, atglistatin *in vitro*.***A*, control or TNF (20 mg/ml) treated 3T3-L1 adipocytes (*B*) control or LPS (100 ng/ml) treated 3T3-L1 adipocytes cocultured with RAW cells were cotreated with vehicle or 10 μM atglistatin for 24 h. FAHFA levels were measured *via* targeted LC-MS. n = 3 per group. ∗*p* < 0.05; ∗∗*p* < 0.01; compared with control; $*p* < 0.05; $$*p* < 0.01; and $$$*p* < 0.001; compared with LPS. *t* test). Error bars represent SD. ATGL, adipose triglyceride lipase; FAHFA, fatty acid hydroxy fatty acid; HETE, hydroxyeicosatetraenoic acid.

ATGL is commonly known for its lipolytic activity on triglycerides to remove one fatty acid to form diacylglycerols and free fatty acids (23). With FAHFAs, ATGL has shown to function as a lipase that can release FAHFAs from FAHFA-containing triglycerides, and, more surprisingly, ATGL was discovered to have a previously unknown transacylase activity that moves an acyl chain from a triglyceride to a hydroxy fatty acid to produce the FAHFA ester bond (16, 17). To ensure that inflammation-induced FAHFA production was not occurring *via* a different mechanism, we looked to measure FAHFA levels in cell culture in the presence and absence of an ATGL inhibitor. Furthermore, to simply the experiment, we did not add linoleic acid to these experiments and focused on PAHSA and oleic acid esters of hydroxystearic acid levels. Adipocytes were treated with TNF to promote inflammation, while cocultures were treated with LPS. To inhibit ATGL we utilized the ATGL inhibitor, atglistatin (24). These experiments demonstrated that inflammation-induced FAHFA production is dependent on ATGL, and atglistatin reduces the production of these lipids (Fig. 4), with the effects being more evident and reaching statistical significance in the more abundant FAHFAs, PAHSA and POHSA.

### FAHFA production as a potential mechanism to inactivate proinflammatory hydroxy fatty acids

**Figure 5:**
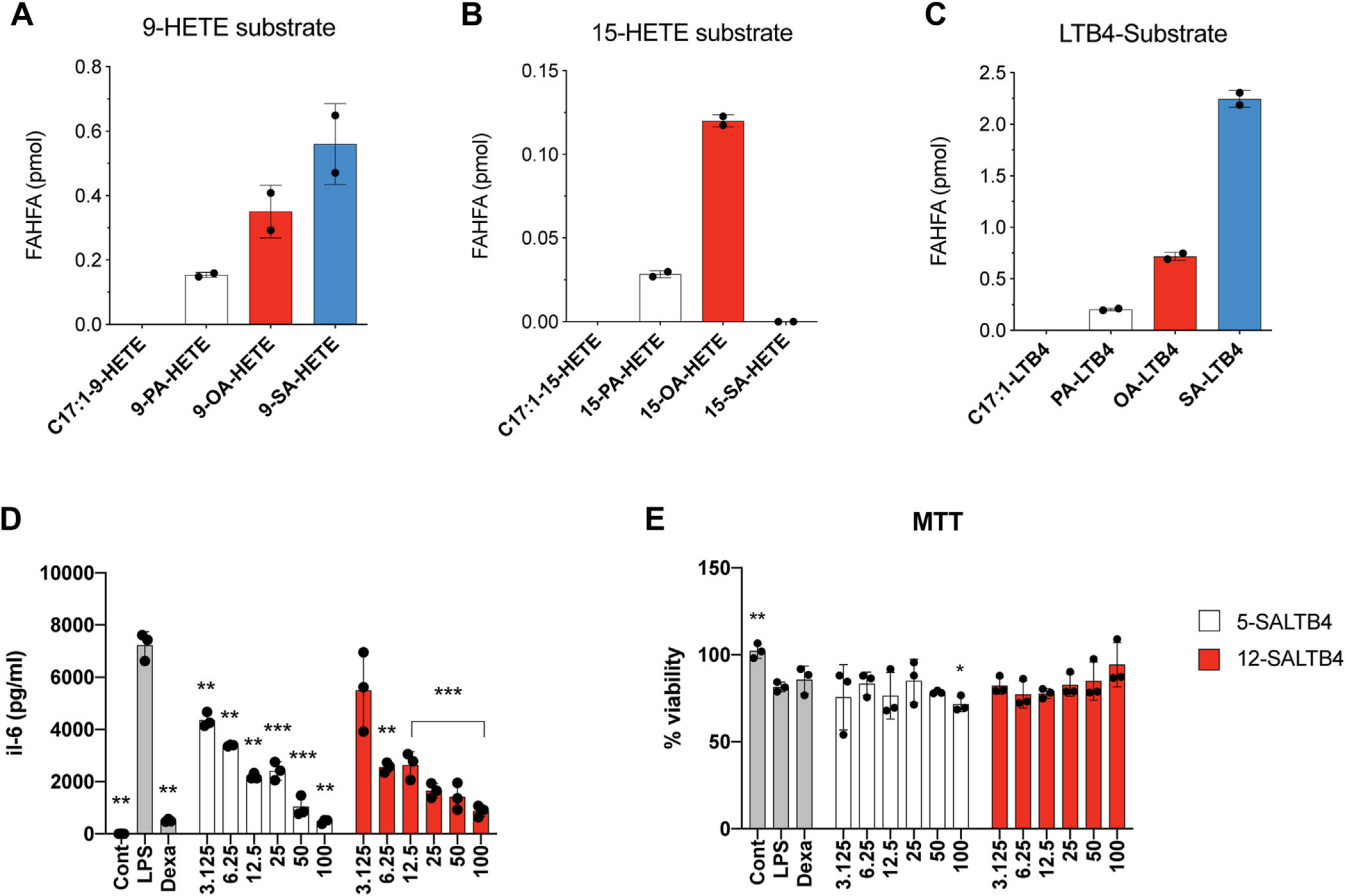
**Other oxylipins contribute to synthesis of FAHFAs with anti-inflammatory activity *in vitro*.** RAW cells were treated with 100 μM (*A*) 9-HETE, (*B*) 15-HETE, or (*C*) LTB4 along with 20 mM C17:1 for 2 h. HSA and HODE-derived FAHFAs were measured *via* targeted LC-MS (n = 2 per group). RAW cells were treated with vehicle (control), or 100 ng/ml LPS with or without 5 μM dexamethasone or indicated concentrations of 5-SALTB4 or 12-SALTB4 for 24 h. *D*, IL-6 levels were measured *via* ELISA. *E*, cell viability was inferred using MTT assay (n = 3 per group. ∗*p* < 0.05; ∗∗*p* < 0.01; and ∗∗∗*p* < 0.001, compared with LPS. *t* test). Error bars represent SD. HODE, hydroxyoctadecadienoic; HETE, hydroxyeicosatetraenoic acid; FAHFA, fatty acid hydroxy fatty acid; LTB4, leukotriene B4; SALTB4, stearic acid LTB4.

HODEs are oxidation products that are often used as markers for inflammation. For instance, 13-HODE is found in psoriatic legions, providing a clinical example of this concept (25). The conversion of HODEs to FAHODEs suggests that a wide range of hydroxy fatty acids are FAHFA precursors, and we wondered if this could extend to bioactive hydroxy fatty acids. Like HODEs, HETEs are disease markers (26), but these lipids also have cellular bioactivities (27, 28). Leukotrienes, which include the bioactive LTB4, are disease causing hydroxy fatty acids that are targets of the pharmaceutical industry (29, 30, 31, 32). Treatment of cells with HETEs or LTB4 resulted in the production of HETE and LTB4 variants of FAHFAs (Fig. 5, *A–C*). There are two hydroxy groups on LTB4 at the 5- and 12-position and we chemically synthesized stearic acid 5-LTB4 (5-SALTB4) and 12-SALTB4. As with other FAHFAs, we found that the SALTB4s had modest anti-inflammatory activities and were nontoxic to cells (Fig. 5, *D* and *E*). We did not detect HETE and LTB4-derived FAHFAs in the adipose tissue of LPS-treated mice, but this could be due to their amounts or differential temporal regulation. Nevertheless, the finding of FAHODEs indicates a pathway from inflammation-associated hydroxy fatty acids and FAHFAs, which should be explored further as a means to regulate inflammation.

## Discussion

Several FAHFAs are anti-inflammatory, glucoregulatory, and cytoprotective lipids (1, 4, 8, 9, 10, 11, 33). Hence, how FAHFAs are regulated under physiological and pathophysiological conditions is imperative to understanding FAHFA biology as well as discovering novel pathways and targets for treatment of human disease. FAHFAs are regulated physiologically during feeding fasting cycles (1) and upon cold exposure (18) in mice, and under pathophysiological conditions in insulin resistance in mice and human (1, 34, 35). This study identifies a new stimulus, inflammation for FAHFA upregulation. It also introduces a possible aspect of FAHFA biology where inflammation leads to upregulation of FAHFAs which in turn may modulate inflammation by neutralizing proinflammatory lipids, such as HETEs and leukotrienes.

While FAHFAs were upregulated in PGWAT and SQWAT, there was no upregulation observed in BAT. It is unclear why FAHFA levels were not upregulated in BAT at time points analyzed. LPS injection leads to activation of inflammatory genes in BAT (36) as well as PGWAT (37) although the extent of inflammatory gene upregulation in BAT can be limited compared to WAT (38). It is possible that response to inflammatory insults in BAT utilizes different pathways in lipid metabolism compared to PGWAT or SQWAT. It is also possible that there is still FAHFA upregulation in BAT but is transient and/or occurs at different time points than we measured. Indeed, we observed different kinetics for FAHFA upregulation in BAT in response to acute cold exposure (Fig. S5). Overall, this study discovered a novel pathway to regulate FAHFAs and similarly a new way to think about hydroxy fatty acid metabolism in cells. These findings are exciting but additional work is necessary to fully understand the role of these pathways in endogenous lipid metabolism. For instance, additional studies looking at the absolute amounts of HODEs and FAHODEs over time *in vivo* would reveal the importance of this pathway in HODE metabolism. Additionally, experiments in animal models where HODE production or FAHODE production is modulated can be used to ascertain whether FAHODEs serve any other role than that of a marker of inflammation.

An additional finding is the need for crosstalk between macrophages and adipocytes for FAHFA production. While TNF could spur adipocytes to upregulate FAHFA production, cocultures of macrophages and adipocytes resulted in a more pronounced FAHFA upregulation. This is likely due to additional or specific factors from the adipocyte, though CM experiments suggest that the factor(s) are either transient or too dilute upon diffusion. Future efforts to identify these factor(s) would be of interest since they could play a significant role in adipose inflammation, which leads to insulin resistance, for example.

In closing, we discovered strong endogenous regulation of FAHFAs, specifically FAHODEs upon acute inflammation. This finding led to additional insights such as the importance of crosstalk between the immune system and adipocytes *via* unknown factors and also led to *in vitro* experiments that suggest that proinflammatory hydroxy fatty acids—HETEs and leukotrienes—might be modified to regulate their activity. Though additional work is required to properly test and support these ideas.

## Experimental procedures

### Animal studies

C57BL/6J mice were purchased from JAX or bred in-house. Mice were housed in 12-h light/dark cycle and fed chow diet (PicoLab, 5053). All animal procedures were approved by Institutional Animal Care and Use Committees of the Salk Institute.

### Materials

LPS (O111:B4) was purchased from Sigma (L4391) for cell culture studies and from Enzo Life Sciences (ALX581012L002) for *in vivo* studies. Atglistatin (SML1075), 12-HSA (219967), heptadecenoic acid (H8896), and dexamethasone (D4902) were purchased from Sigma. (±)9-HODE (38400), (±)13-HODE (38600), (±)9-HETE (34400), (±)15-HETE (34700), Prostaglandind2 (12010), linoleic acid (90150) were purchased from CAYMAN. LTB4 (sc-201043) was purchased from Santa Cruz Biotechnology. 3-(4,5-dimethylthiazol-2-yl)-2,5-diphenyltetrazolium bromide (MTT) (475989) was purchased from EMD Millipore. IL-6 (431304) and TNF (430904) ELISAs were from Biolegend. Recombinant mouse TNF-alpha (410-MT) was from R&D Systems. 13-LAHODE, 5-, and 12-SALTB4 were synthesized as described (39, 40).

### Cell culture

RAW 264.7 cells were maintained in RPMI with 10% fetal bovine serum (FBS) at 37 °C, 5% CO_2_. 3T3-L1 preadipocytes were maintained in high glucose Dulbecco's modified Eagle's medium (DMEM) with 10% bovine calf serum at 37 °C, 10% CO_2_.

### 3T3-L1 differentiation

3T3-L1 cells were seeded on day −4. Once they were confluent on day −2, Medium was refreshed with DMEM containing 10% bovine calf serum. On day 0, medium was switched to DMEM with 10% FBS with differentiation cocktail (5 μg/ml insulin, 1 μM dexamethasone, and 500 μM 3-isobutyl-1-methylxanthine). Cells were cultured for 3 days, then switched to DMEM with 10% FBS containing 5 μg/ml insulin, and medium was refreshed every 2 days. Day 8 to 12 differentiated adipocytes were used for experimentation.

### FAHFA biosynthesis assay

RAW 264.7 cells were seeded in 6-well plates. At approximately 80% confluency, cells were washed with cell culture medium and treated with 100 μM of 12-HSA, 9-HETE, 15-HETE, LTB4, 9-HODE, 13-HODE, and 20 μM C17:1 for 2 h. Each treatment replicate was performed in 1 well. Cells were washed with cold PBS 3 times and collected with cell scraper and snap-frozen. Cell pellets were lysed *via* sonication and total lipids were extracted in 1:1:2 ml of PBS:methanol:chloroform including 5 pmol 13C4-9PAHSA as internal standard. Results were normalized per well.

### Anti-inflammatory assay with RAW 264.7 cells

RAW 264.7 cells were seeded 20 K per well in 96-well plate and incubated overnight. Next day, cells were washed with culture medium and treated with 100 ng/ml LPS, with or without 5 μM dexamethasone, varying concentrations of 13-LAHODE, 5-SALTB4, and 12-SALTB4 for 24 h in 200 μl volume. One hundred microliters of CM was collected, and IL-6 ELISA was performed according to manufacturer’s instructions to assess cytokine secretion. Then, MTT assay was performed.

### MTT assay

At the end of anti-inflammatory assay, 8 μl of 4 mg/ml MTT solution was added on top of cells and incubated for 45 min at 37 °C. MTT crystals were dissolved with 100 μl of isopropanol with 0.04 N HCL. Absorbance at 570 and 630 nm (background) was read with a Biotek Synergy plate reader. Background absorbance was subtracted from absorbance at 570 and percent viability was calculated with control cells set to 100% viability.

### LPS, TNF, and CM treatment in cell culture and lipid extraction

Day 8 to 10 3T3-L1 adipocytes in 6-well or 10 cm plates were washed with culture medium and treated with 20 mg/ml TNF or 100 ng/ml or 1 μg/ml LPS for 2 or 24 h. For coculture experiments, 3T3-L1 adipocytes were washed with cell culture medium and 100 K or 500 K RAW 264.7 cells were added on 3T3-L1 adipocytes in 6-well or 10 cm plates, respectively, at t = −4 h. At t = 0, cells were treated with 100 ng/ml LPS for 2 or 24 h. For CM treatments, 400 K RAW cells/well were seeded in 6-well plates overnight. Next day, cells were treated with 100 ng/ml LPS for 24 h. CM was collected, spun at 2000*g* for 5 min, and filtered with 0.22-micron filter. One milliter of CM + 1 ml of DMEM with 10% FBS was added on differentiated adipocytes and incubated for 2 or 24 h. In experiments involving C17:1 and linoleic acid treatments, adipocytes were treated with 20 ng/ml TNFα for 22 h. Then, 100 μM of linoleic acid or C17:1 for 2 h. In experiments to assess ATGL role in FAHFA regulation, 10 μM atglistatin or dimethylsulfoxide was added at the same time with LPS or. For lipid extraction, cells were washed with cold PBS 3 times and lysed in 900 μl PBS *via* sonication. Protein concentrations were measured for normalization. Total lipid was extracted on 0.9:0.9:1.8 ml PBS:methanol:chloroform with addition of 5 pmol of 13C4-9-PAHSA as internal standard. Samples were vortexed for 15 s and centrifuged at 2600*g* for 6 min for phase separation. Lower phase was collected and dried for FAHFA analysis without SPE mediated enrichment.

### *In vivo* anti-inflammatory assay

Twelve-week-old, male mice were injected with vehicle (50% PEG400, 0.5% Tween-80, 49.5% water) or 10 mg/kg 13-LAHODE i.p. at t = −1 h. All mice were injected with 0.15 mg/kg of LPS i.p. at t = 0. 24-week-old, male mice were injected with vehicle (50% PEG400, 0.5% Tween-80, 49.5% water) or 10 mg/kg 9-PAHSA i.p. at t = −0.5 h. All mice were injected with 1 mg/kg of LPS i.p. at t = 0. Eighteen-week-old, male mice were injected with vehicle (50% PEG400, 0.5% Tween-80, 49.5% water) or 10 mg/kg 13-OAHODE i.p. at t = −1 h. All mice were injected with 0.15 mg/kg of LPS i.p. at t = 0. Plasma samples were collected at t = 0, 1 h, 2 h, and 6 h, and mice were sacrificed at the end of the study. Plasma TNFα and IL-6 levels were measured *via* ELISA according to manufacturer’s instructions.

### LPS injection of mice and FAHFA extraction

For initial studies, 9-week-old, male, C57BL/6J mice were injected i.p. with 3 mg/kg LPS. GWAT, SQWAT, and BAT were collected at t = 0 prior to injection, t = 8h and 24h. Approximately 100 mg of SQWAT and PGWAT, and 40 mg of BAT were homogenized in 1.5:1.5:3 ml of PBS:methanol:chloroform. 5 pmol of 13C4-9-PAHSA was added during extraction as an internal standard. After homogenization, samples were vortexed for 15s and centrifuged at 2500*g* for 6 min for phase separation. Total lipids in lower phase were collected and dried for FAHFA enrichment using solid phase extraction as published previously (14). Nonesterified FAHFA fractions were dried for further analysis.

### Targeted LC-MS for FAHFA analysis

For tissue analysis, extracted FAHFAs were resuspended in 50 to 200 μl of methanol and analyzed using LC-MS method as previously described (41). Ten microliters of sample was injected to UPLC BEH C18 Column (Waters Acquity, 186002350). Thermo TSQ mass spectrometer *via* multiple reaction monitoring was used for measurement of FAHFAs using the transitions outlined in Table S2. Using Skyline, peak areas were calculated and normalized to the peak areas of the internal standards and the tissue weight. For cell analysis, total lipids were resuspended in 50 μl methanol and analyzed as above. FAHFA levels were normalized with total protein content.

### Cold exposure studies

Seven-month-old C57BL/6J, male mice were transitioned from room temperature to a cold chamber set to 4 °C. SQWAT, PGWAT, and BAT were collected at t = 0 (no cold exposure), t = 2 h, and 24 h upon cold exposure. FAHFAs were extracted as above, and levels were measured by LC-MS.

## Data availability

The representative data are contained within the article. Direct all other data inquiries to A. S.

## Supporting information

This article contains supporting information.

## Conflict of interest

The authors declare that they have no conflicts of interest with the contents of this article.
