# Supplementary material for: Acute inflammation upregulates FAHFAs in adipose tissue and in differentiated adipocytes
Source: J Biol Chem. 2024 Nov 5;300(12):107972. doi: 10.1016/j.jbc.2024.107972 (PMC11647607; doi:10.1016/j.jbc.2024.107972)
Supplement: Supplemental Table S2 [file mmc2.pdf]

Transition	Precursor ion	Product ion	Collision Energy (V)
POHC17:1_transition1	519.5	253.2	29
POHHC17:1_transition3	519.5	283.2	28
PAHC17:1_transition1	521.5	255.2	29
PAHC17:1_transition3	521.5	283.2	28
POHODE_transition1	531.4	253.2	29
POHODE_transition3	531.4	295.2	27
PAHODE_transition1	533.5	255.2	29
PAHODE_transition3	533.5	295.2	27
C17:1HC17:1_transition1	533.5	267.2	29
C17:1HC17:1_transition3	533.5	283.2	28
POHSA_transition1	535.5	253.2	29
POHSA_transition2	535.5	281.2	27
POHSA_transition3	535.5	299.3	27
PAHSA_transition1	537.5	255.2	29
PAHSA_transition2	537.5	281.2	27
PAHSA_transition3	537.5	299.3	27
13C4-PAHSA_transition1	541.5	259.2	29
13C4-PAHSA_transition2	541.5	281.2	27
C17:1_HODE_transition1	545.5	267.2	29
C17:1_HODE_transition3	545.5	295.2	27
OAHC17:1_transition1	547.5	281.2	29
OAHC17:1_transition3	547.5	283.2	28
C17:1-HSA_transition1	549.5	267.2	29
C17:1-HSA_transition2	549.5	281.2	27
C17:1-HSA_transition3	549.5	299.3	28
C17:1_LTB4_transition1	557.4	267.2	29
C17:1_LTB4_transition3	557.4	335.2	28
PAHETE_transition1	557.5	255.2	29
PAHETE_transition3	557.5	319.2	28
LAHODE_transition1	557.5	279.2	29
LAHODE_transition3	557.5	295.2	27
OAHODE_transition1	559.5	281.2	29
OAHODE_transition3	559.5	295.2	27
OAHSA_transition1	563.5	281.2	29
OAHSA_transition3	563.5	299.3	27
C17:1_HETE_transition1	569.5	267.2	29
C17:1_HETE_transition3	569.5	319.2	28
PALTB4_transition1	573.5	255.2	29
PALTB4_transition3	573.5	335.2	28
OAHETE_transition1	583.5	281.2	29
OAHETE_transition3	583.5	319.2	28
SAHETE_transition1	585.5	283.2	29

SAHETE_transition3	585.5	319.2	28
POPGD2_transition1	587.4	253.2	29
POPGD2_transition3	587.4	351.2	27
PAPGD2_transition1	589.5	255.3	29
PAPGD2_transition3	589.5	351.2	27
OALTB4_transition1	599.5	281.2	29
OALTB4_transition3	599.5	335.2	28
SALTB4_transition1	601.5	283.2	29
SALTB4_transition3	601.5	335.2	28
OAPGD2_transition1	615.5	281.2	29
OAPGD2_transition3	615.5	351.2	27
