# Supplementary material for: Acute inflammation upregulates FAHFAs in adipose tissue and in differentiated adipocytes
Source: J Biol Chem. 2024 Nov 5;300(12):107972. doi: 10.1016/j.jbc.2024.107972 (PMC11647607; doi:10.1016/j.jbc.2024.107972)
Supplement: Supplemental Fig. S1–S5 [file mmc3.docx]

**Figure S1.** FAHFAs are formed of fatty acids and hydroxy fatty acids. The isomer is named based on the carbon number where ester bond is formed. Precursor, PA and 9-HSA, for 9-PAHSA is depicted.


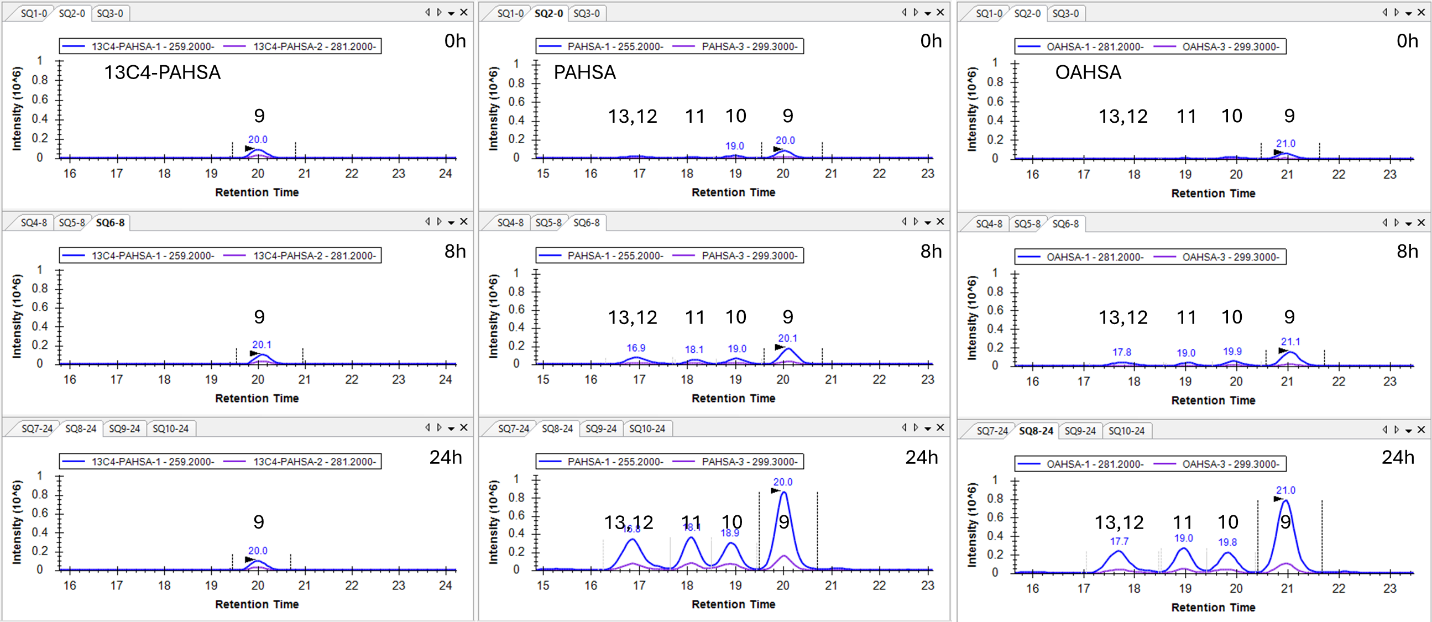


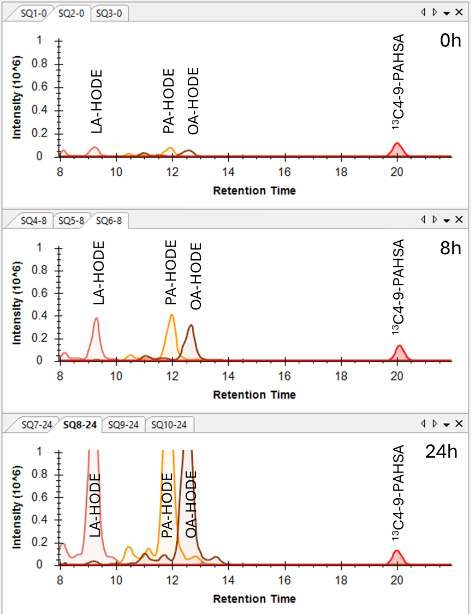


**Figure S2.** Representative LC-MS chromatograms demonstrating the increasing levels of FAHFAs at different time points.


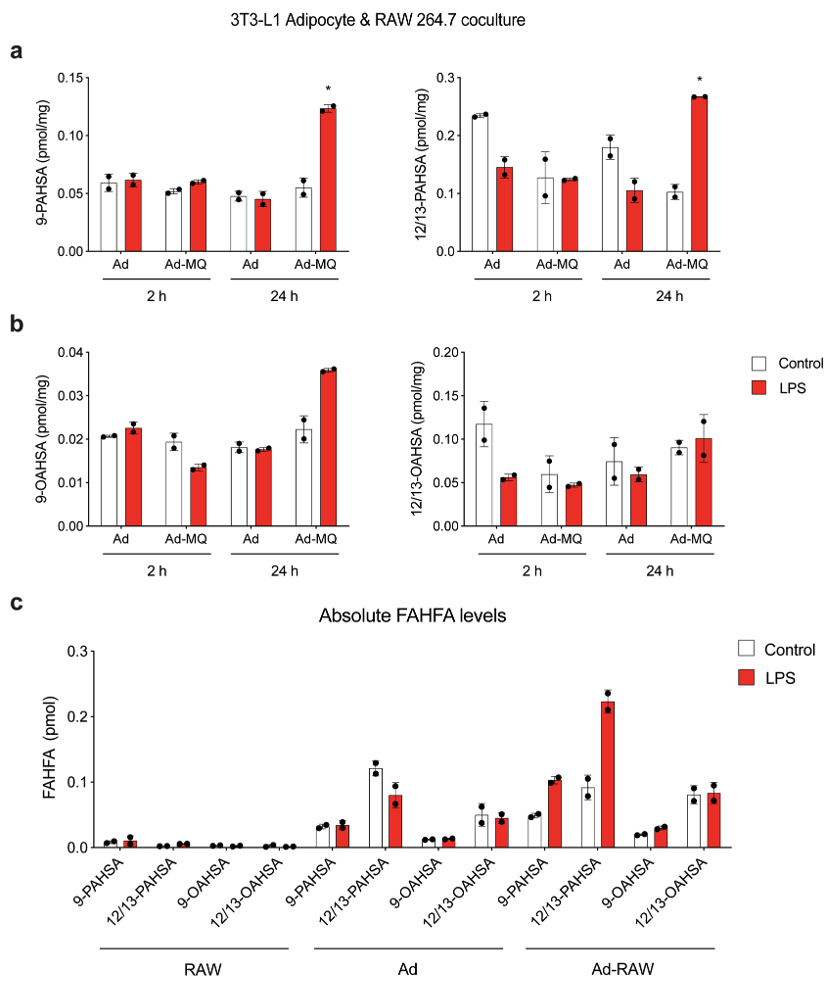


**Figure S3. Inflammation induced FAHFA upregulation in 3T3-L 1 adipocytes co-cultured with RAW cells.** 3T3-L 1 adipocytes co-cultured with RAW cells were treated with 100 ng/ml LPS for 2h or 24h. (a) 9- and 12/13-PAHSA and (b) 9- and 12/13-OAHSA levels were measured using targeted LC-MS. (c) Representation of above data as absolute levels of FAHFAs measured in RAW cells, adipocytes (Ad), or adipocytes-RAW cocultures treated with control media or 100 ng/ml LPS for 24h for side by side comparison (n=2 per group. *, p < 0.05, compared with control. t test). Error bars represent SD.


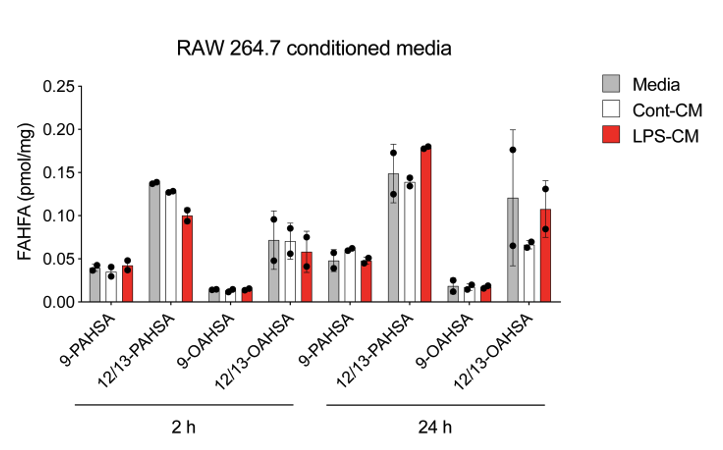


**Figure S4. FAHFA levels in 3T3-L 1 adipocytes treated with RAW conditioned media.**

Adipocytes were treated with regular media or conditioned media from RAW cells treated with control medium or 100 ng/ml LPS for 2h or 24 h. (n=2 per group). Error bars represent SD.


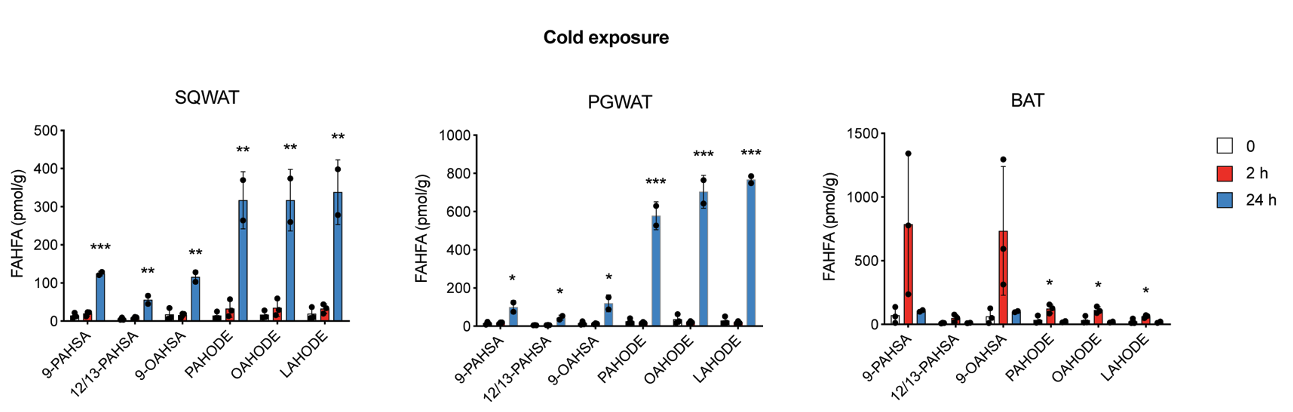


**Figure S5. FAHFAs regulation during cold exposure.** FAHFA levels in SQWAT, PGWAT, and BAT of mice subjected to cold for 0h, 2h or 24h. (n=2-3 per group).
